# Development of ICF Core Sets to standardize assessment of functioning and impairment in ADHD: the path ahead

**DOI:** 10.1007/s00787-013-0496-5

**Published:** 2013-12-14

**Authors:** Sven Bölte, Elles de Schipper, Martin Holtmann, Sunil Karande, Petrus J. de Vries, Melissa Selb, Rosemary Tannock

**Affiliations:** 1 Neuropsychiatric Unit, Department of Women’s and Children’s Health, Center of Neurodevelopmental Disorders (KIND), Stockholm, Sweden; 2 Division of Child and Adolescent Psychiatry, Stockholm County Council, Stockholm, Sweden; 3 LWL-University Hospital for Child and Adolescent Psychiatry, Psychotherapy and Psychosomatics, Ruhr University Bochum, Hamm, Germany; 4 Learning Disability Clinic, Department of Pediatrics, Seth G.S. Medical College & K.E.M. Hospital, Mumbai, India; 5 Division of Child and Adolescent Psychiatry, University of Cape Town, Cape Town, South Africa; 6 ICF Research Branch in cooperation with the WHO Collaborating Centre for the Family of International Classifications in Germany (at DIMDI), Nottwil, Switzerland; 7 Swiss Paraplegic Research (SPF), Nottwil, Switzerland; 8 Neurosciences and Mental Health Research Program, The Hospital for Sick Children, University of Toronto, Toronto, Canada

**Keywords:** Neurodevelopmental disorders, Assessment, Children and youth, Psychiatry, Mental health, Health care

## Abstract

In the study of health and quality of life in attention deficit/hyperactivity disorder (ADHD), it is of paramount importance to include assessment of functioning. The International Classification of Functioning, Disability and Health (ICF) provides a comprehensive, universally accepted framework for the description of functioning in relation to health conditions. In this paper, the authors outline the process to develop ICF Core Sets for ADHD. ICF Core Sets are subgroups of ICF categories selected to capture the aspects of functioning that are most likely to be affected in specific disorders. The ICF categories that will be included in the ICF Core Sets for ADHD will be determined at an ICF Core Set Consensus Conference, wherein evidence from four preliminary studies (a systematic review, an expert survey, a patient and caregiver qualitative study, and a clinical cross-sectional study) will be integrated. Comprehensive and Brief ICF Core Sets for ADHD will be developed with the goal of providing useful standards for research and clinical practice, and to generate a common language for the description of functioning in ADHD in different areas of life and across the lifespan.

## Background

Attention deficit/hyperactivity disorder (ADHD) is a neurodevelopmental disorder of complex origin, with an estimated worldwide prevalence of 5.3 % [1]. It is associated with a multitude of increased risks, such as specific learning disorders, school drop-out, low self-esteem, depression, anxiety, delinquent behavior, substance abuse, and under-employment [2–4]. The World Health Organization (WHO) International Classification of Diseases (ICD-10) [5] defines ADHD (F90.0) along the three behavioral domains of inattention, hyperactivity and impulsivity. To justify a diagnosis of ADHD, a childhood onset of persistent symptoms and their presence across a range of situations and contexts is required. In addition, the general definition of a mental disorder must be met, which includes individual suffering or injury, threat to one’s own or others’ health, or limitations to the individual’s capacities. The latter is in line with the WHO’s definition of health as a state of complete physical, mental and social well-being and not merely the absence of disease or infirmity [6]. That is, a person’s health is defined by more than the absence or presence of a disorder or disease. Particularly, adaptive functioning and quality of life (QoL) must be considered within a comprehensive evaluation of health.

A rich body of literature shows that individuals with ADHD of all ages experience functional impairments in many areas of everyday living, such as underachievement in school, difficulty in finding and keeping employment, and poor social relationships [4, 7]. Besides functional impairments, QoL has been considered as an additional outcome in several studies of ADHD. Definitions, possibilities and challenges relating to QoL in child mental health are discussed in a review by the ADHD European Guidelines Group, in which they provide clinically and scientifically relevant arguments for the value of QoL as an outcome measure in ADHD [8]. In a review on QoL in childhood ADHD, Danckaerts et al. [9] concluded that parents of children with ADHD consistently rate the QoL of their children below the population norm. Children with ADHD also rated their QoL to be lower than that of their peers without ADHD, although less consistently than their parents. For QoL in adult ADHD, a review by Agarwal et al. [10] also observed lower QoL in individuals with ADHD compared to the general population.

Interestingly, the Danckaerts et al.’s review [9] suggested that it often seems difficult to distinguish between the symptoms of ADHD (e.g., impulsivity, hyperactivity) and their associated functional impairments (e.g., behavioral difficulties) when using diagnostic instruments to assess the former or the latter. However, Üstün [11] argued that a clear differentiation between ADHD symptoms on the one hand, and their impact on functioning on the other, is crucial for a better understanding of how ADHD as a neurodevelopmental disorder can diversely influence individual functioning. In addition, detailed information about level of functioning and specific difficulties and strengths is indispensable when creating tailored individual intervention plans and evaluating their effectiveness. Although the multiaxial classification of a disorder in ICD-10 [12]/DSM-IV-TR [13] includes a global rating of psychosocial and functional problems, the Global Assessment of Functioning Scale (GAF), and DSM-5 introduces a new system for assessing functional impairment independent of diagnostic symptoms, the WHO Disability Assessment Schedule (WHODAS 2.0), these tools do not provide a comprehensive profile of an individual’s level of functioning in face of a certain disorder. Both the GAF and the WHODAS essentially generate a single summary score of functioning between 0 and 100 (full disability to no disability; reverse scaling on the WHODAS). While the WHODAS, as compared to the GAF, offers additional severity scoring and interpretation opportunities on a range of items and six daily living domains, it remains rough in its coverage of factors relevant to functioning and is not tailored for any more specific condition. A complementary system for a more comprehensive and standardized assessment of functioning in the context of certain environmental and personal factors is necessary to derive clear indications for treatment. In addition, such a system would improve diagnostic and outcome research, inter- and trans-disciplinary communication, and the calculation of related costs for support in ADHD. With the objective to serve these purposes, the WHO designed the International Classification of Functioning, Disability and Health (ICF) [14].

### The WHO international classification of functioning, disability and health (ICF)

**Fig. 1:**
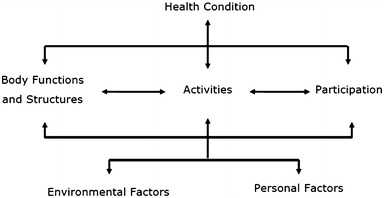
The integrative biopsychosocial model of functioning, disability and health

The WHO has developed the ICF [14], among other reasons, to standardize the assessment of functioning in relation to health conditions. The concept of functioning is introduced explicitly in the biopsychosocial model which forms the framework of the ICF [15]. According to this model, an individual’s level of functioning is the outcome of a complex interaction between a health condition, body functions and structures, activities and participation, environmental factors, and personal factors. The interaction among these components is dynamic and bidirectional; changes in one component may influence one or more of the other components. This interactive model is shown in Fig. 1. QoL as such is not explicitly included in the biopsychosocial ICF model, but McDougall and colleagues [16] argue that it can be considered an implicit part of it, in terms of an outcome of the interaction between the different components of the model.

According to the ICF model, health condition is a disorder or disease that a person is diagnosed with, for instance ADHD (ICD-10, F90.0: hyperkinetic disorder). Body functions are the physiological functions of body systems, while body structures are anatomical parts of the body, such as organs and limbs. For instance, corresponding categories in the case of ADHD include impulse control (b1304) in the body functions component and structure of brain (s110) in the body structures component. An activity is the execution of a task or action by an individual, and participation represents the involvement in a life situation. Examples of activities and participation relating to ADHD are focusing attention (d160) and informal relationships with friends (d750). Environmental factors make up the physical, social and attitudinal environment in which people live. With regard to ADHD, relevant environmental factors include medication (e1101) and special education and training services (e5853). Lastly, personal factors are the particular background of a person’s life and living, and they comprise features of the individual that are not part of a health condition [14], such as gender, race, age, other health conditions, fitness, lifestyle, habits, coping styles, social background, education, profession, past and current experience. Personal factors are not specifically coded in the ICF, owing to their variability among cultures, and ICF users may assess and describe them in a manner that is suitable for their use. Examples of personal factors that may be relevant to ADHD are age, gender, SES. Environmental factors as well as personal factors can act as facilitators to the person, when they improve functioning or even eliminate disability entirely. Similarly, these factors can act as barriers, when they produce or increase a person’s level of disability (for a detailed example of how the biopsychosocial model of the ICF can be applied to ADHD, see Üstün [11]).

Derived from the ICF in 2007, the ICF Children and Youth version (ICF-CY) [17] was designed to capture the particular situation of the developing child by adding categories and expanding on descriptions that specifically take into account the fact that (a) the child needs to be viewed in the context of the family; (b) the nature and forms of participation change dramatically from dependent relationships in infancy to complex life situations in adolescence; (c) along with developmental changes in participation, the nature and number of environments change as well; and (d) lags in emergence of functions or acquisition of skills may reflect developmental delay rather than impairments or stable limitations [18]. Even though ADHD can remit in adolescence, the disorder is often chronic and associated impairments persist across the lifespan in most cases [19]. To adequately capture the impact of ADHD on individuals across the lifespan, the ICF-CY (including all ICF categories) will be used in the project presented here.

The ICF and the ICF-CY are part of the WHO’s Family of International Classifications, developed to provide a comprehensive and universally accepted framework to classify the experience of health in individuals as well as populations. Even though the ICD-10 is the most widely used classification system, there is a growing interest in the use of the ICF and ICF-CY [referred to as “ICF(-CY)” from now on] in international health care, particularly with regard to physical disabilities [18, 20–22]. Additionally, there is increasing ICF(-CY) awareness and usage in psychiatry [22, 23].

**Fig. 2:**
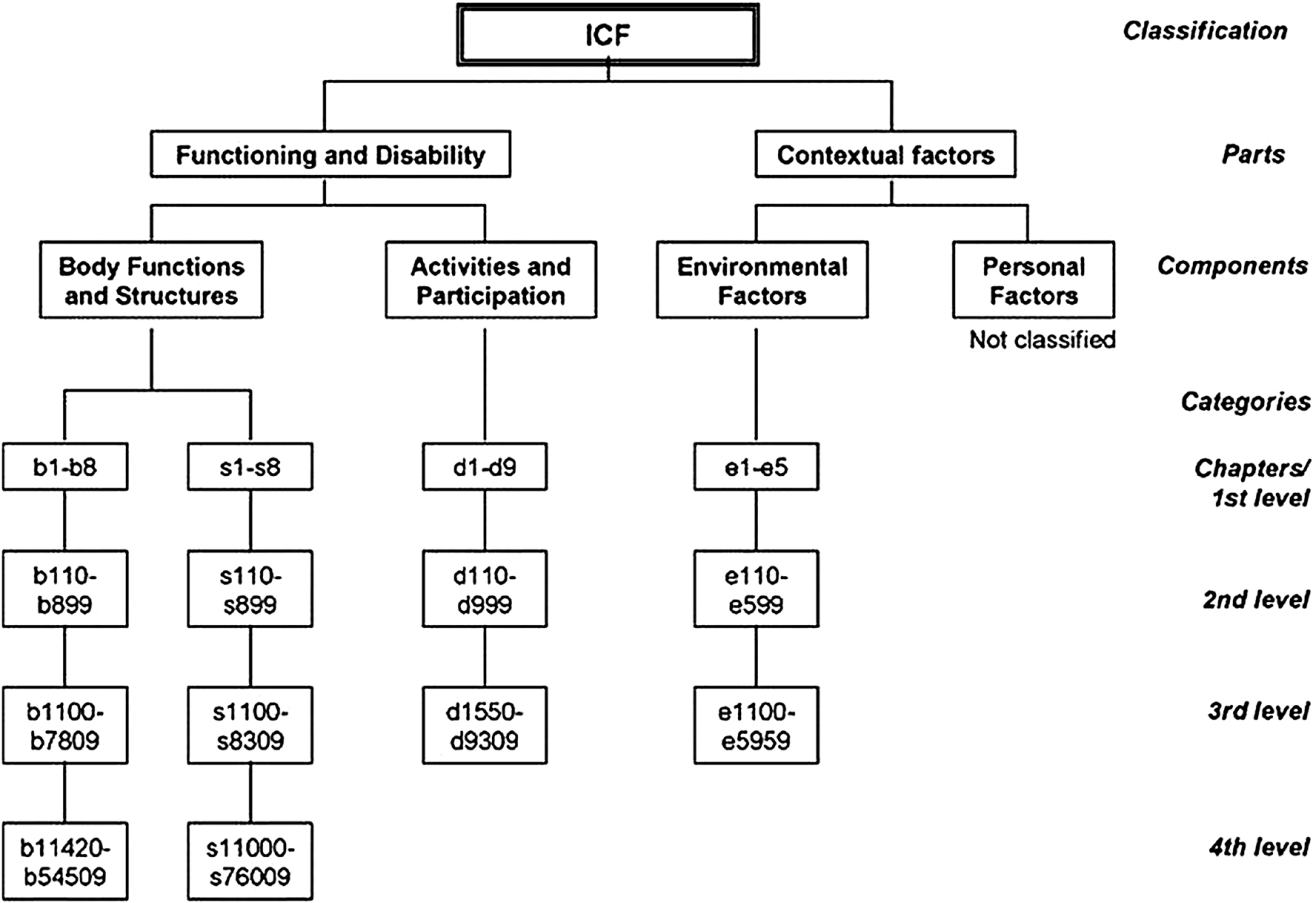
Hierarchical structure of the ICF. ICF categories each have a unique code, which is built up of a prefix and a numeric code. Codes included in the different levels are shown for each component. The prefix is a letter indicating the component of which the category is a part: *b* body functions, *s* body structures, *d* activities and participation, and *e* environmental factors. The numeric code starts with the first level or chapter number (one digit), followed by the second level (two digits), and the third and fourth level (one digit each)

The ICF(-CY) is hierarchically structured and has two parts, each consisting of two components (see Fig. 2). In turn, each component consists of categories, which describe the content of each component and are the units of classification. The categories are organized into four levels, containing progressive levels of detail in their descriptions. The first-level categories are called chapters and provide a general overview of the different areas of functioning covered in the ICF(-CY). For example, chapters in the body functions component include mental functions and voice and speech functions, and the activities and participation component includes chapters such as communication, self-care, and domestic life. The chapters consist of second, third and sometimes fourth-level categories [12].

The hierarchical structure of the ICF(-CY) can be seen in the following ADHD example from the body functions component:

- First-level chapter: b1 mental functions
- Second-level category: b130 energy and drive functions
- Third-level category: b1304 impulse control

The ICF(-CY) consists of over 1,400 categories providing a comprehensive and exhaustive classification of an individual’s functioning. However, the extensiveness of the ICF has proven to be both its strength and its weakness, for in its current full version it is too extensive for practical use in daily clinical practice. To address this issue the ICF Research Branch, a partner of the German Collaboration Centre of the WHO Family of International Classifications and the WHO Classification Terminology and Standards (CTS) team, with the support of the International Society of Physical and Rehabilitation Medicine (ISPRM), the World Confederation for Physical Therapy (WCPT), the World Federation of Occupational Therapists (WFOT), and the International Society for Prosthetics and Orthotics (ISPO), initiated the development of so called ICF “Core Sets” [24, 25]. ICF Core Sets are shortlists of ICF categories that are considered most relevant to individuals with a certain health condition, and on which assessment tools (e.g., questionnaires, interviews, observation scales, checklists) [26, 27] can be based. These ICF Core Sets are formed by means of a rigorous qualitative and quantitative, scientifically structured process. The established process involves a large selection of researchers and clinicians, as well as clients and their caregivers, from all over the world; which ensures the universal applicability of these official ICF Core Sets. To date, ICF Core Sets have already been developed for 24 conditions (see Table 1), two of which for mental health conditions, namely depression and bipolar disorder.

**Table 1:** ICF Core Sets for health conditions already developed and related publications

Mental health conditions	
Depression	[32]
Bipolar disorder	[33]
Other health conditions	
Neurological conditions	
Multiple sclerosis (MS)	[34]
Neurological Conditions for acute care	[35]
Neurological Conditions for early post-acute care	[36]
Spinal cord injury (SCI)	[37]
Traumatic brain injury (TBI)	[38]
Cardiovascular and respiratory conditions	
Cardiopulmonary Conditions for acute care	[39]
Cardiopulmonary Conditions for early post-acute care	[40]
Obstructive pulmonary diseases	[41]
Obesity	[42]
Diabetes mellitus	[43]
Stroke	[44]
Chronic ischaemic heart disease	[45]
Cancer	
Head and neck cancer	[46]
Breast cancer	[47]
Musculoskeletal conditions	
Acute inflammatory arthritis	[48]
Ankylosing spondylitis	[49]
Chronic widespread pain	[50]
Musculoskeletal Conditions for acute care	[51]
Musculoskeletal Conditions for early post-acute care	[52]
Osteoporosis	[53]
Osteoarthritis	[54]
Low back pain	[55]
Rheumatoid arthritis	[56]
Other health conditions	
Hearing loss	[57]
Vertigo	[58]
Inflammatory bowel diseases	[59]
Sleep	[60]
Hand conditions	[61]
Diverse situations	
Geriatric Patients	[62]
Vocational rehabilitation	[63]

### Aim of this project

The aim of this project is to develop official ICF Core Sets for ADHD and to provide a standardized method to classify functioning in individuals with a diagnosis of ADHD (referred to as “clients” from now on) at all ages. These ICF Core Sets for ADHD are unique in that they are developed based on a rigorous scientific protocol and as an official effort in collaboration with the WHO and the ICF Research Branch in cooperation with the WHO Collaborating Centre for the Family of International Classifications in Germany (at DIMDI). This process ensures that multiple international perspectives are captured and that a comprehensive picture of functioning is formed, and that the universal character of the ICF(-CY) is preserved by involving experts, clients and clinicians with various professional backgrounds and from all six WHO regions. Moreover, this project adds to the existing research by developing ICF Core Sets for ADHD that are applicable to children, adolescents and adults alike. This makes it possible to follow the functional development of individuals with ADHD using the compatible tools across the entire lifespan. The objective of this paper is to describe and preview the development process of these ICF Core Sets for ADHD.

## Methods

### Development of ADHD ICF Core Sets

**Fig. 3:**
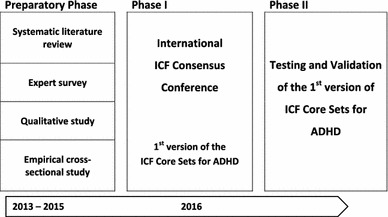
ICF Core Set for ADHD development process

This project is conducted in conformity with the ethical principles of the Declaration of Helsinki. All appropriate study-related documents will be presented to the corresponding Ethics Committees for review and approval, and informed consent will be collected from participants in the qualitative and the empirical cross-sectional study. ICF Core Sets will be developed in a three-phase process: (1) preparatory phase, (2) phase I (international consensus conference resulting in the first version of the ICF Core Sets for ADHD); and (3) phase II (validation and testing of the ICF Core Sets). The different phases of the development process are presented in Fig. 3.

### Preparatory phase

During the preparatory phase information on functioning in ADHD based on the ICF and ICF-CY will be gathered, and based on this information a pre-selection of relevant ICF(-CY) categories will be made. The pre-selected categories will then be presented to an international panel of ADHD experts at the consensus conference to help them make an informed decision about which of the pre-selected ICF(-CY) categories should be included in the ICF Core Sets. The preparatory phase consists of four scientific studies, each addressing the selection of relevant ICF(-CY) categories from a different perspective:

#### Systematic literature review (researcher perspective)

A systematic review of studies published since 1982 will be performed (1) to identify outcome measures in ADHD research, and (2) to link the concepts in these measures to the ICF(-CY). After selection of relevant studies from multiple databases (Medline/PubMed, PsycINFO, ERIC, CINAHL), the data collection process will consist of three consecutive steps: (1) the parameters used in selected studies will be identified; (2) the items of retrieved parameters and their underlying concepts will be specified; and (3) the concepts will be linked to the categories of the ICF(-CY) using established linking rules [28]. Absolute and relative frequencies of the outcome measures and the ICF(-CY) categories to which they are linked will be reported.

#### Expert survey (opinion leader perspective)

To gather the expertise of an international pool of ADHD opinion leaders regarding aspects of functioning that are relevant to ADHD, an internet-based survey will be performed. The pool will include experts (≥5 years of experience working with individuals with ADHD) from various disciplines [coaches, nurses, occupational therapists, physical therapists, physicians (neurologists and psychiatrists), psychologists, psychotherapists, speech and language therapists, social workers and teachers] and from each of the six WHO regions (Europe, the Americas, Africa, Eastern-Mediterranean, South-East Asia, and Western Pacific). The experts to be included in the survey will be selected by identifying as many key opinion leaders in the field of ADHD as possible with the help of international professional organizations and societies, journal editorial boards, and other networks. Selected experts will be invited to take part in an internet-based survey. Participating experts will be asked to name the concepts they consider most relevant with regard to functioning in ADHD across the lifespan. From their statements a stratified random sample will be drawn (*n* = ~200) and retrieved concepts will be linked to ICF(-CY) categories using established linking rules [28]. The output of this study will be a list of relevant ICF(-CY) categories with corresponding absolute and relative frequencies.

#### Qualitative study (client and other perspective)

Focus groups with clients, caregivers, teachers, and spouses will be conducted to explore and understand which aspects of functioning in ADHD are important to them. A set of questions covering the components of the biopsychosocial model (e.g., “If you think about your daily life, what are your problems?”) will be employed to guide the discussion in groups of 4–6 people under supervision of a moderator. To ensure the universal applicability of the ICF Core Sets for ADHD, study centers from each of the six WHO regions will be approached for participation in this study. Based on previous ICF Core Set studies, approximately six focus groups are expected to be held per study center. Since the focus groups will include clients of all ages, the questions will be rephrased in an age-appropriate manner according to the age of the focus group participants. Where an individual is unable to take part in a focus group due to the severity of the condition, an individual interview will be performed. The written transcription of the focus group discussions and individual interviews will be analyzed to identify concepts of functioning important to the clients. These concepts will then be linked to ICF(-CY) categories, using established linking rules [28]. Like the expert survey, the output of this study will be a list of relevant ICF(-CY) categories with corresponding absolute and relative frequencies.

#### Empirical cross-sectional study (clinical perspective)

A cross-sectional study will be conducted to identify the most common problems in functioning experienced by individuals with ADHD, as reflected by the clinician’s perspective. A total of 200 children, adolescents and adults with ADHD will be selected to take part in this study, preferably from study centers in each of the six WHO regions. Based on information from client records, observations and semi-structured interviews, clinicians will rate the functioning of their clients using a case record form (CRF) based on the extended ICF Checklist 2.1a [29]. The extended ICF Checklist 2.1a comprises ICF(-CY) categories that address the most common problems experienced by clients in clinical practice, extended with categories specific to ADHD that will be selected based on results from the systematic literature review and the expert survey. In addition, a measure of QoL will be included in the checklist to get a complete picture of the clients’ health situation according to the WHO definition of health [6]. Clinicians’ rating of their clients’ functioning will be analyzed and absolute and relative frequencies of the ICF(-CY) categories relevant to ADHD will be reported.

### Phase I

The information collected during the preparatory phase will be presented at an international consensus conference, planned to be held in 2016, where a group of 21–25 experts in the field of ADHD from all WHO regions will follow a formal decision-making process to arrive at a consensus on the ICF(-CY) categories to be included in the Comprehensive and Brief ICF Core Sets for ADHD. The Comprehensive Core Set will include the ICF(-CY) categories that reflect the entire spectrum of typical problems that clients may encounter at all ages, and is thereby suitable for a comprehensive and interdisciplinary assessment of functioning in ADHD. Based on decisions to be made by an international steering committee, one or more Brief Core Set will be derived from the Comprehensive Core Set and will capture the essence of a client’s functioning. It is intended to be the starting point for basic clinical documentation, as well as the minimal standard for describing functioning in ADHD in clinical and epidemiological studies. The consensus conference will start with an introduction to the ICF(-CY), the process of developing ICF Core Sets, and the results from the preparatory studies. This introduction will then be followed by a structured decision-making process, involving alternating work group sessions and plenary sessions, in which the participants discuss and vote on the categories to be included in the Comprehensive ICF Core Set. After the Comprehensive ICF Core Set is decided, a ranking process will follow to select the categories for the Brief ICF Core Set.

### Phase II

Phase II will consist of validating the ICF Core Sets for ADHD. Specific aims of the Phase II study include (1) to verify whether categories included in the ICF Core Sets for ADHD describe the entire spectrum of typical problems encountered by clients from all over the world; (2) to identify possible relevant categories missing from the ICF Core Sets for ADHD; and (3) to examine the applicability of the categories of the ICF Core Sets in different contexts, for different purposes, and from different perspectives. An international, cross-sectional, multicenter validation study with individuals diagnosed with ADHD will be conducted to study the content validity and feasibility of the ICF Core Sets for ADHD.

## Discussion

In this paper, we described the rationale and proposed scientific process for the development of ICF Core Sets for ADHD. The ICF Core Sets for ADHD will be intended to be used as a guide for making recommendations for the practical use of the ICF(-CY) in the context of ADHD, by providing a selection of the most relevant ICF(-CY) categories that facilitate the description of a client’s functioning. In the project described in this paper, both Comprehensive and Brief Core Sets will be developed. The Comprehensive Core Set can provide clinicians with a basis for a thorough and interdisciplinary assessment of functioning, for the formulation of intervention goals, and for the evaluation of progress in treatment. The Brief Core Set can be used when only a brief assessment of functioning is necessary. With a smaller number of categories they capture the essence of functioning of clients.

### Aims and uses of ICF Core Sets for ADHD

ICF Core Sets for ADHD provide an overview of the aspects of functioning that should be assessed to get a complete picture of the level of functioning of an individual with ADHD. This overview can be translated into various assessment instruments, such as questionnaires, rating systems, structured interviews, observation scales, etc.—depending on the needs and wishes of the user. Neither the Comprehensive nor the Brief Core Sets will be exhaustive, and users are free to add ICF(-CY) categories to enhance assessment of functioning for their specific purposes. Because the assessment instruments based on the ICF Core Sets for ADHD will be translated from the ICF(-CY) categories, this means it will also be possible to translate them back to these categories. This in turn means that assessment instruments based on the ICF Core Sets for ADHD will be easy to compare, even if they are used in different disciplines or countries.

The complex nature of ADHD requires a multidisciplinary and multilevel assessment and intervention approach to improve functioning and QoL for individuals with this disorder. The ICF Core Sets for ADHD can meet this need by providing a common basis for communication across different disciplines in ADHD research and clinical practice. In addition to enhancing multi- and trans-disciplinary communication, the ICF Core Sets can provide scientists with terminology and definitions of functioning that are universally applicable and understandable irrespective of country and cultural borders, thus facilitating international studies.

Since ICF Core Sets for ADHD will outline “what to measure”, rather than “how to measure”, they will not become ready-to-use instruments for measuring functioning. After Phase II, tools for assessment of functioning will need to be derived from the ICF Core Sets for ADHD, and these tools will need to be validated and implemented to ensure that the framework of the ICF Core Sets will be widely applied. Applications of the ICF framework include quantifying the effect of ADHD on functioning, and evaluating the effectiveness of interventions. Moreover, ICF Core Sets for ADHD can be used to rate the content validity of already existing measures and thereby to select appropriate instruments specifically relevant to ADHD. We envision that the wide application of ICF Core Sets for ADHD in research and clinical practice will lead to improved knowledge about functioning in ADHD, which in turn will lead to interventions that improve functioning and QoL for individuals with ADHD.

### Developing ICF Core Sets for ADHD: challenges and opportunities

In preparing the development process of the ICF Core Sets for ADHD, we encountered certain questions and challenges that needed to be considered prior to the preparatory studies. How to best tackle these issues was discussed and determined by an international Steering Committee (SC) during a meeting that took place in Stockholm in May 2013. This SC consists of ADHD experts from different professions and WHO regions and guides the development process of ICF Core Sets for ADHD. One of these issues discussed during the meeting is the fact that ADHD is a developmental condition that often persists across the lifespan, and that symptoms as well as needs may vary in nature and severity with age [19].This presents a challenge for the development of ICF Core Sets for ADHD covering the lifespan, as the categories that are relevant to describe functioning may also vary for different age groups. The SC decided that one Comprehensive ICF Core Set for ADHD should be developed which is applicable across the lifespan, while two or more Brief ICF Core Sets for ADHD could be developed specific to the developmental stages, if needed. The four preparatory studies will be decisive in determining if several Brief ICF Core Sets will be necessary in ADHD and for which developmental stages if deemed necessary. The results from the preparatory studies will be grouped according to developmental stage: childhood, adolescence and adulthood. This is preferred over grouping according to age, because the ages for developmental stages differ across countries and cultures. Depending on the ICF-CY categories included in the different groups, the SC will be able to decide on the adequate number of meaningful Brief ICF Core Sets for ADHD, and the developmental stages to which they should apply. These Comprehensive and Brief ICF Core Sets for ADHD will then be determined by a group of independent experts during the international consensus conference.

Another well-known challenge in ADHD research, which was determined by the SC, is the question of how to handle psychiatric comorbidity and its impact on functioning in individuals with ADHD. The co-occurrence of other neurodevelopmental and psychiatric disorders is the rule rather than the exception in ADHD [30, 31]. This is a significant possible confounding factor when generating the ICF Core Sets for ADHD, and needs to be considered carefully when trying to distinguish between functional impairments resulting from ADHD, and comorbid disorders, respectively. However, the ICF Core Sets for ADHD are designed to be representative of the majority of the population with this specific disorder. Therefore, it was decided that any functional impairment that is commonly experienced by individuals with ADHD, whether or not it results directly from the disorder, should be included in the ICF Core Sets for ADHD in order for them to be representative tools.

In conclusion, the consensus conference will provide us with a first version of the ICF Core Sets for ADHD, and subsequent testing and validation will be needed before a standardized and universally accepted tool for the classification of functioning in ADHD will be available. The project described in this paper forms the crucial first step towards the achievement of this tool.
